# Changes and Correlations Between the Width and Height of the Hard Palate During Rapid Maxillary Expansion with a Printed Tooth-Borne Expander

**DOI:** 10.3390/healthcare13212756

**Published:** 2025-10-30

**Authors:** Mirela Georgieva, Emanuel Emiliyanov, Greta Yordanova

**Affiliations:** Department of Orthodontics, Faculty of Dental Medicine, Medical University of Sofia, 1432 Sofia, Bulgaria; mirela.georgieva@fdm.mu-sofia.bg (M.G.); emanuelemilianov@gmail.com (E.E.)

**Keywords:** rapid maxillary expansion, CBCT, growing patients, molar angulation, tooth-borne, orthodontics, skeletal change

## Abstract

Background/Objectives: The nasomaxillary complex is a compound anatomical structure in which the correlation between changes in palatal width and height has been poorly investigated. Methods: A three-year prospective study with 28 patients treated with printed expanders was conducted. Measurements on CBCT images were performed before and after treatment; the width and height parameters were measured on a coronal slice of a CBCT image at the level of the first molars and upper first molar inclination relative to the palatal plane. Results: A significant difference was found between the values measured before and after treatment, including an increase in the measured width parameters and a statistically significant decrease in palatal height (0.75 ± 0.97). The mean change in the upper molar inclination was not significant (tooth 16: 0.60 ± 6.42; tooth 26: 2.19 ± 4.51). The regression analysis did not establish a significant correlation between the expansion of the maxillary occlusal width and palatal height change or a significant correlation between the increase in the upper molar inclination and palatal height change. Conclusions: The use of a printed tooth-borne expander causes predictable and reproducible skeletal changes. It is a feasible treatment option, allowing for precise screw positioning to achieve bodily movement of the teeth and decrease the change in molar inclination.

## 1. Introduction

Transverse deficiency of the maxilla is invariably associated with changes in the craniofacial skeletal structures of the nasomaxillary complex and the surrounding soft tissues. Various treatments for maxillary transverse deficiency (MTD) have been performed in orthodontic practice for many years. Rapid maxillary expansion (RME) with tooth-borne and bone-borne appliances has proven to be effective and has become an established treatment option. With the development of CAD/CAM technologies in orthodontics, personalised digitally designed expanders are increasingly used for better therapeutic results. They mitigate the adverse effects of vestibular crown inclination in the molars [1].

Transverse deficiencies in the nasomaxillary complex modify the anatomy, which has an unfavourable effect on the development and function of the upper respiratory tract [2,3,4].

MTD is often associated with posterior crossbite (unilateral or bilateral), crowding of the teeth, and a narrow and high palatal vault. MTD can be isolated or combined with other sagittal or vertical dentofacial deformities that clinically mask the anomalies in the transverse dimension [5].

RME is a contemporary and widely accepted treatment method for MTD in patients during growth or late adolescence. Ossification of the midpalatal suture continues throughout adolescence (14–15 years) or even until its end (18 years of age), allowing for successful rapid expansion with tooth-borne support [6,7,8,9]. The tooth-borne maxillary expander separates the midpalatal suture, which has not yet reached full ossification in growing individuals. This is caused by lateral displacement of the two alveolar processes of the maxilla [10,11].

The expectation with contemporary digitally designed metal printed tooth-borne expanders (DDMPTBE) is minimal movement of the supporting teeth, which was often observed with previous conventional versions of expanders [12,13,14,15,16]. Despite these findings, the authors describe that RME with deciduous supporting teeth and traditionally manufactured RME with tooth-borne support achieve an increase in the bone dimensions of the nasomaxillary complex [17]. In their analysis, Weissheimer et al. confirmed that RME achieves an increase in all transverse measurements of the upper jaw [16]. The expansion has a triangular pattern, with less skeletal effects than dental effects. This indicates that the magnitude of dental expansion exceeds that of skeletal expansion, a phenomenon attributable to the influence of vestibular molar inclination [16]. It is natural for orthodontists to gravitate toward expanders that reduce the effect of molar inclination; namely, expanders with bone-borne support. To minimise these side effects and increase skeletal changes induced by RME in growing patients, the Hybrid Hyrax Expander (HHE) was introduced [18]. However, HHE has deleterious effects, such as inflammation, pain, appliance breakage or distortion, and asymmetrical expansion [19]. These effects led to the use of DDMPTBE to attempt to minimise the molar inclination effects like HHE, without bone anchorage and its deleterious effects.

The growth of digital planning capabilities for orthodontic appliances has the potential to enhance their functionality. It enables the implementation of elements that address adverse effects and facilitate pre-planning of the biomechanical consequences of tooth movements. Software for the digital design of appliances allow for precision in every detail of the expander (position of the screw, bands covering the anchorage teeth, and rigid connecting structures, among others) [20,21,22,23]. This level of detail in planning has been demonstrated to reduce deleterious clinical effects and improve treatment outcomes.

The skeletal and dental changes from RME in contemporary orthodontics are measured on CBCT images. CBCT demonstrates high accuracy in assessing not only bone structures but also the pharyngeal airway volume, cross-sectional area of the upper airways, facial soft tissues, and other parameters [4,24]. The development of dental software allows for the automatic segmentation of bone structures and parts of the upper airway using artificial intelligence (AI) [25,26]. Morphological studies with CBCT have been shown to surpass traditional two-dimensional (2D) cephalometric studies in terms of analysing maxillary deficiencies. The measurements can be performed at different levels: dental arch, alveolar width, bone width, nasal base width, and other values [4,27].

The nasomaxillary complex is a compound anatomical area that is closely related to the nearby bone and soft tissue structures. Changes in one of its characteristics inevitably lead to changes in other characteristics [28]. The extent of the correlation between changes in the width and height of the hard palate has not been sufficiently studied and described. Few studies have assessed the impact of transverse skeletal discrepancy (TSD) on the severity of orthodontic deformity and the relationship between sagittal and vertical skeletal patterns. The data in the literature on changes in the transverse width of the maxilla and their dependence on the inclination of the upper first molars, which inevitably accompanies the process of maxillary expansion, are insufficient. These reasons led the authors to investigate this problem in orthodontic patients treated with DDMPTBE. Therefore, the aim was to study the craniofacial anatomical changes in the individual characteristics of the maxilla and the relationships between them when using a DDMPTBE.

## 2. Materials and Methods

A three-year prospective clinical study was conducted based on the results of digital measurements on CBCT images of 28 patients treated with a DDMPTBE by the team. All patients were diagnosed with MTD associated with unilateral or bilateral posterior crossbite. The most common etiological factor for this deformity was functional breathing disorders (impaired nasal breathing).

The mean age of the study group was 12.43 ± 2.62 years. The age group range was from 8 to 16 years. Of the participants included in the sample, 12 (42.9%) were boys and 16 (57.1%) were girls.

**Figure 1:**
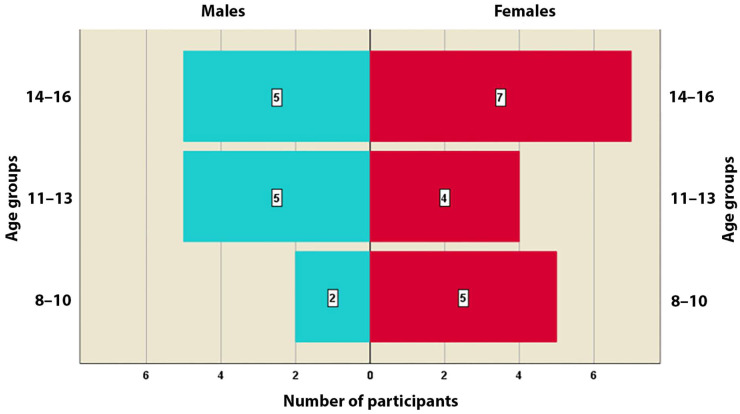
Distribution of the study participants by sex and age groups.

The age of the patients includes prepubertal growth, active growth, and residual growth. The growth stage was assessed using cervical vertebrae maturation (CVM) method. The patients were divided into three age groups: ages 8–10 years (7 patients—2 males and 5 females); ages 11–13 years (9 patients—5 males and 4 females) and ages 14–16 years (12 patients—5 males and 7 females). The distribution is shown in Figure 1.

According to the type of dentition, patients were divided into three dentition groups: early mixed, late mixed, and permanent dentition. The largest relative proportion of patients (75.0%) had permanent dentition, only 7.1% had late mixed dentition, and 17.9% of the included participants had early mixed dentition.

The values of the ≮ANB were used to determine the skeletal class. ≮ANB is used to determine the relationship between the maxilla and the mandible and define the skeletal relationship class, and is calculated as the difference between the ≮SNA and ≮SNB.

≮SNA determines the position of the maxilla and is formed by the Sella–Nasion line and point A (the deepest point of concavity on the front of the maxilla).

≮SNB determines the position of the mandible and is formed by the Sella–Nasion line and point B (the deepest point of concavity on the front of the mandible).

Based on their skeletal characteristics, the patients included in the study were divided according to the skeletal class into the following groups:

- Skeletal class I—≮ANB = [0–4°], with a relative share of 60.7%;
- Skeletal class II—≮ANB > 4°, with a relative share of 21.4%;
- Skeletal class III—≮ANB < 0°, with a relative share of 17.9%.

In order to determine the type of vertical skeletal growth, patients were classified according to ≮MP/SN.

≮MP/SN is used to assess the vertical relationship between the skull and the mandible and to define the vertical skeletal growth of the lower facial third. ≮MP/SN is formed by the intersection of the Sella–Nasion line and the mandibular plane. A higher ≮MP/SN indicates a longer lower facial third (“longer face”) and a lower ≮MP/SN indicates a shorter facial third (“short face”).

Patients were grouped into the following categories based on the values of the angle:

- Normodivergent type—≮MP/SN = [29–35°], with a share of 32.1%;
- Hypodivergent type—≮MP/SN < 29°, with a share of 32.1%;
- Hyperdivergent type—≮MP/SN > 35°, accounting for 35.7%.

### 2.1. Ethics Statement

The clinical procedures were carried out according to the guidelines of the World Medical Association’s Declaration of Helsinki and the Ministry of Health for Good Clinical Practice. This study was approved by the Ethics Committee of KENIMUS, Approval Code: 7995/07.10.2024. Consent was obtained from patients or their guardians for treatment involving the use of RME.

### 2.2. Clinical Considerations

For both males and females, the appropriate time for treatment is during mixed dentition and early permanent dentition (before and at the start of the peak of adolescent growth), and the efficacy of the treatment is likely to vary between the sexes depending on growth activity. The growth stage is conventionally determined in practice using CVM, in cases of doubt, CBCT images of the maxilla are analysed to determine the maturation of the palatal suture before orthodontic treatment is planned.

### 2.3. Inclusion Criteria

The criteria used for the inclusion of patients in this study were as follows:

- Patients must be diagnosed with MTD and with uni- or bilateral posterior crossbite and the absence of other severe craniofacial malformations;
- Complete documentation (including whole-skull CBCT scans at the initial phase and the end of the treatment phase with RME);
- Informed consent after the treatment plan (non-extraction treatment) was presented, discussed, and accepted, as certified by the signature of the patient (or parent);
- Strictly followed the treatment protocol, without missed control appointments;
- Patients must be without agenesis and previous orthopaedic or orthodontic treatments.

### 2.4. Clinical Protocol for the Application of a DDMPTBE

Treatment was carried out after a complete diagnostic protocol, which included CBCT imaging. A treatment plan was devised, involving an initial phase of RME. After informing the patients and their parents (guardians) and obtaining their consent, orthodontic treatment was conducted.

The maxillary expansion of all the patients was the first phase of the main treatment. The second phase was with fixed techniques during the period of active growth and pubertal development.

**Figure 2:**
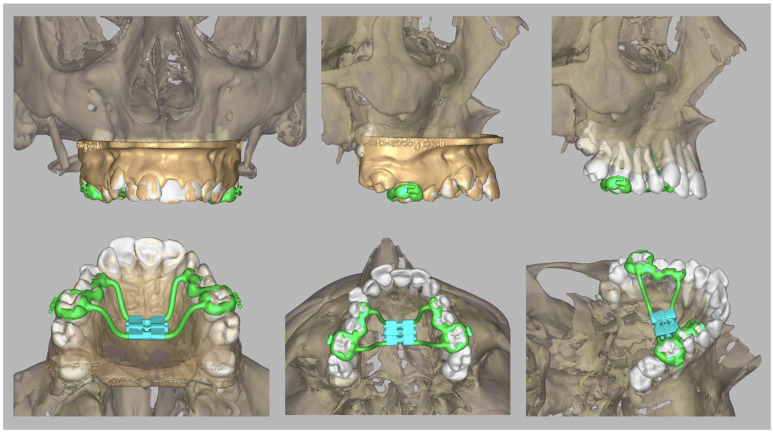
Digital design and plan of an expander and superimposition of the intraoral scan and CBCT image using Exocad DentalCAD 3.0 Galway software.

The device used for RME of the maxilla was planned and manufactured individually for each patient based on a 3D model obtained after intraoral scanning and superimposition with the CBCT image. The software and the CBCT volumetric image used to design the appliance facilitate the precise placement of the expansion screw relative to the axes of the anchorage teeth, thereby enabling the optimal planning of the anchorage system that encompasses the posterior teeth. This allows the placement of the screw to be closest to the level of the centre of resistance (CR) of the first molars and perpendicular to their axial planes. The CR is considered the fundamental reference point for controlled tooth movement. For the upper first molar, the CR is considered slightly apical to the area of the root’s trifurcation (Figure 2).

The goal of digital planning is to minimise the inclination of the molars and the deleterious effects that result from it. The design of the appliance was made with Exocad software (Exocad DentalCAD 3.0 Galway) and was printed with CAM technology from the Co–Cr alloy by laser sintering the metal. The appliance was bonded to the posterior anchorage teeth in the maxilla.

The following activation protocol for the RME is the one that the research team uses in their daily practice. The patient and accompanying parent were trained to activate the appliance. The activation protocol included approximately 20–28 screw activations, depending on the severity of the MTD. The screw was activated once a day at the same time in the evening. The activation protocol was the same for all of the included patients: 1/4 turns (0.25 mm) per day until the desired transverse occlusal relationship was achieved between the upper and lower posterior teeth—slight overcorrection of the normal transverse occlusal relationship. When the activation and expansion were complete, the patients underwent a 3-month retention period.

The patient monitoring and follow-up appointments were scheduled as follows: one appointment was scheduled on the day of the last screw activation during the activation phase to determine if the desired transversal relationships were achieved; if they were not achieved, a set of additional screw activations was determined and a new appointment was set on the last activation day. After the desired transversal relationship was achieved, the retention phase began.

In the retention phase, the appointments were scheduled once a month for a total of three months. The purpose of these appointments was to monitor the stability of the result and the condition of the appliance. During the third appointment at the end of the third month, the expander was debonded, a follow-up CBCT image was obtained and the second phase of the treatment began. The data from the CBCT image were then used to perform the necessary measurements. Patient compliance was high because the appointments took little clinical time and the time between the appointments did not interfere with the accompanying parent’s schedule.

### 2.5. Reference Planes, Variables, and Measurement Methods

All of the cephalometric and CBCT measurements were obtained twice by an orthodontic specialist at 2-week intervals, and the mean values of the two measurements were used for the analysis. Intraclass correlation coefficients (ICCs) were used to assess the intra-examiner reliability for the cephalometric and CBCT measurements and ranged from 0.95 to 0.98 and from 0.94 to 0.97, respectively.

**Figure 3:**
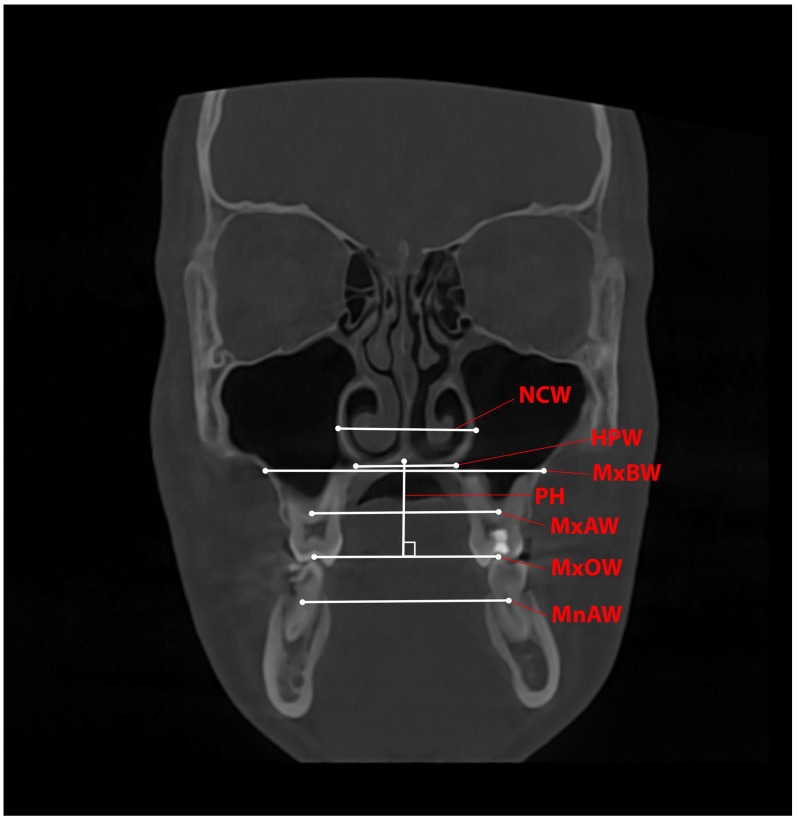
Transverse and vertical measurement of skeletal variables on a CBCT coronal slice. The corresponding parameter is marked in red letters, and its location is marked with a thin red line. The white lines with dots indicate where the parameter is measured.

Measurements of the transverse and vertical skeletal changes were performed on a coronal slice of the CBCT image at the level of the CR of the upper first molars before and after expansion. The values were recorded in mm (Table 1 and Figure 3).

To determine the level of TSD of the maxilla, the TDI was used. The index was introduced by Roh et al. [4] in one of their studies and represents the difference between MxAW and MnAW. A discrepancy is defined when the TDI < −2.5 mm, and there is no discrepancy when TDI > −2 mm.

**Figure 4:**
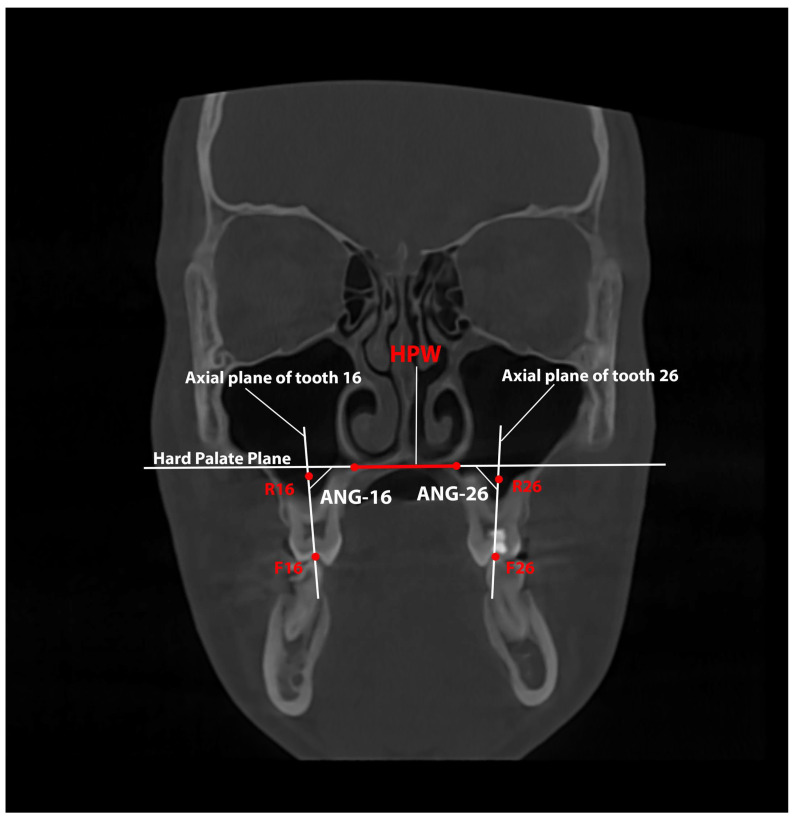
Assessment of the inclination of the upper first molars relative to the plane of the hard palate measured on a CBCT coronal slice. The Hard Palate Width (HPW) is indicated with the red line and the corresponding plane is indicated in white. The red dots indicate the points (R16, R26, F16, and F26) that define the axial plane of the upper first molars (represented by the two vertical white lines).

Measurements of the inclination of the right (ANG-16) and left (ANG-26) upper first molars relative to the HPW were also made. The measurements were performed before and after RME and were measured in degrees (Table 2 and Figure 4).

### 2.6. Statistical Methods

The data were entered and processed using the statistical packages IBM SPSS Statistics 27.0.1.0. and Excel Office 2021. A significance level of *p* < 0.05 was accepted for rejecting the null hypothesis.

## 3. Results

A significant difference was found between the values of the six variables examined in the nasomaxillary complex (MxOW, MxAW, MxBW, NCW, HPW and PH) before and after treatment. While increases in the measured values (width) of five of the variables were observed, there was a statistically significant decrease in PH, which is clinically observed as a reduction in the height of the hard palate. Table 3 shows the mean values and their standard deviations for the analysed variables before and after RME.

Table 4 shows the extent of the change resulting from the treatment. The most significant increase in the transverse dimension is at the occlusal plane level (MxOW—3.64 mm) and the base of the alveolar ridge (MxAW—3.68 mm). Evidently, a transverse expansion in the basal area of the maxilla is also observed, but to a much lesser extent (MxBW—1.59 mm). Proceeding in the cranial direction, the expansion diminishes, with an NCW of 1.40 mm measured at the nasal cavity and an HPW of 1.19 mm measured at the hard palate. Only the palatal height shows a decrease of 0.75 mm. This confirms the working hypothesis that the expansion of the maxilla and maxillary dental arch is accompanied by a decrease in the height of the hard palate. The reduction in expansion size in the cranial direction confirms the above-mentioned triangular expansion pattern.

As demonstrated in Table 5, the mean change in the inclination of the upper molars was not statistically significant (for tooth 16, the mean change was 0.600; for tooth 26, the mean change was 2.190). Furthermore, the standard deviation for tooth 16 was 6.420, and that for tooth 26 was 4.510. The minimum and maximum values for the parameter in question were deemed to be significant. However, these parameters were measured in individual patients. The wide angulation range obtained is related to the occlusal blockages from the unilateral posterior crossbites. The occlusal blockage acts as an additional anchorage for one of the upper molars, while the contralateral upper molar receives a higher force level, which results in its increased vestibular inclination, thus resulting in deviations from the mean values.

Judging by the mean values, there is relative stability with minimal molar inclination in the area of the first molars during maxillary expansion. This statement was supported by the mean values of MxOW and MxAW, where the roots of the first molars are actually located. Both variables, as shown in Table 4, have almost identical change (MxOW—3.64 mm and MxAW—3.68 mm). This suggests an almost bodily displacement of the upper first molars that is attributed to the digital planning and placement of the expansion screw at the level of the CR of the upper first molars, minimising the potential vertical deformation of the maxilla.

As expected, the palatal height (PH) decreases due to the rapid expansion of the high palatal vault (−0.75 mm). Despite the clinically observed relation between maxillary expansion and a reduction in the palatal vault height, the regression analysis using the curve estimation procedure did not establish a significant correlation between the size of expansion in the MxOW and changes in PH. It also failed to establish a significant correlation between the increase in the angulation of the upper first molars (ANG-16 and ANG-26) and changes in PH.

**Figure 5:**
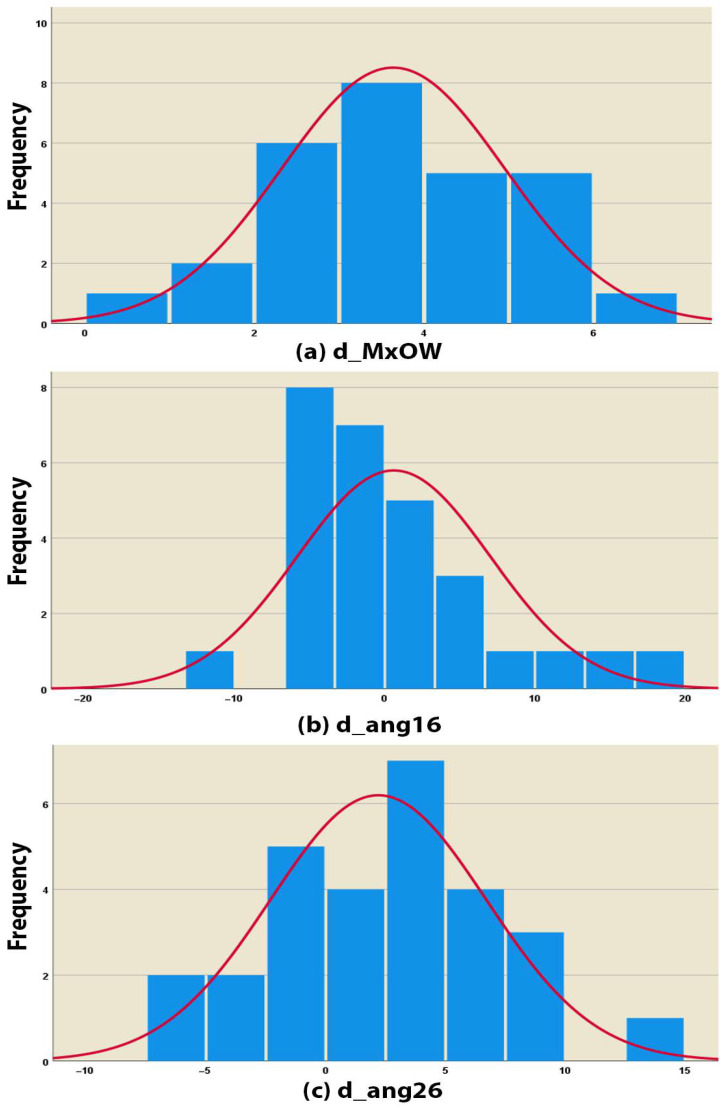
Frequency distributions of the following parameters: (**a**) d_MxOW—Shapiro–Wilk *p* = 0.738; (**b**) d_ang16—Shapiro–Wilk *p* = 0.104; (**c**) d_ang26—Shapiro–Wilk *p* = 0.846.

A statistically significant regression relationship was found between the increase in the upper molars’ inclination (ANG-16 and ANG-26) and the change in MxOW. The normal distribution of the three variables enabled multiple linear regression analysis to be applied (Table 6 and Figure 5). According to the analyses, the relationship is described by the following equation (R^2^ = 0.465, *p* < 0.001):

**d_MxOW** = **3.262** + **0.118**×**d_ang16** + **0.138**×**d_ang26**

where d_MxOW is the change in maxillary occlusal width due to the angulation of the upper first molars d_ang16 and d_ang26. The coefficient of determination (R^2^) shows that the model describes 46.5% of the variance in the dependent variable. According to the standardised Beta coefficients, d_ang16 has the greatest influence, followed by d_ang26.

**Figure 6:**
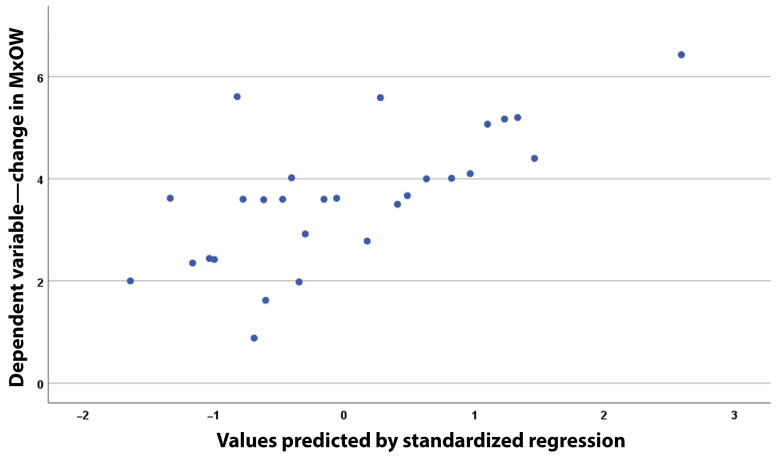
Scatter plot showing the actual and predicted values from the regression model based on the values presented in Table 6.

Figure 6 shows a scatter plot of the actual versus predicted values from the regression model. The high degree of correspondence is confirmed by the clustering of the points into a well-formed ellipse, with a diagonal intersecting the abscissa, and the high correlation coefficient (R = 0.682).

The established and described linear relationship confirms that the compensatory vestibular inclination of the upper first molars has a favourable effect on the achieved expansion at the dental arch level.

## 4. Discussion

The present study aimed to evaluate skeletal and dentoalveolar changes induced by DDMPTBE in mixed and early permanent dentition. In this study, three-dimensional CBCT images were utilised to plan the position of the expansion screws as well as the overall orthodontic treatment. As the results suggest, the digital planning of the screw position attributed to the bodily movement of the upper first molars, giving them an advantage over the conventional expanders. The metal-printed DDMPTBE’s rigidity also contributed to bodily movement, enabling forces to be transferred directly onto the palatal suture without loss due to bending of the structure, as observed with conventional expanders. The DDMPTBE’s bonding procedure to the teeth is fairly easy, as with all tooth-borne expanders, in contrast to the complex and lengthy protocols for planning and positioning palatal screws and the risks involved when using HHE.

The present study draws evidence based on the findings of research conducted with two software programs to determine the skeletal results of expander treatment, as performed by Sfondrini et al. [29]. In the aforementioned study, the authors assessed the effectiveness of both programs in the three-dimensional cephalometric analysis. Research conducted on CBCT is more informative than research conducted on two-dimensional images, due to the possibility of analysing different planes [30,31].

Caldas et al. conducted a study on the changes in the floor of the nasal cavity along the palatal suture [32]. Their findings revealed a gradual decrease in linear measurement dimensions towards the fronto-nasal suture, with values of approximately 1.3 mm, 0.8 mm, and 0.4 mm in the anterior sections, and 1.4 mm, 0.7 mm, and 0.3 mm in the posterior sections. This is understandable given that, when viewed coronally, the suture opening is triangular in shape with the apex pointing towards the frontonasal suture, consistent with the triangular expansion pattern obtained in the present study.

Colino-Gallardo et al. [33] analysed changes achieved with a conventional expander and found that the nasal width (NCW) increased by 1.28 ± 0.64 mm and maxillary width (MxBW) increased by 2.79 ± 1.48 mm, which are very close to results of the current study. Garib et al. conducted a study with conventional Hyrax and found an increase in the NCW of 1.11 mm and in the MxBW of 0.99 mm, as well as an increase of 2.26 mm with HHE [34]. The changes in MxBW reported in the presented study are higher (1.59 mm) than those reported by Garib with a conventional appliance (0.99 mm). Therefore, the more precise planning of the screw position and the rigidity of the structure in the DDMPTBE increases its skeletal efficiency compared to the conventional type. The same authors [34] reported a mean inclination change for the first molars of 7.080 in the group treated with conventional Hyrax, while Colino-Gallardo et al. [33] cited a mean change in the inclination of the molars of 5.62 ± 3.20° for the right molar and 4.74 ± 2.22° for the left molar, which is significantly higher than 0.60 ± 6.420 for tooth 16 and 2.19 ± 4.510 for tooth 26 found in the current study using the DDMPTBE (Table 3). These differences confirm the greater stability exhibited by the monolithic skeletal structure of printed expanders, in comparison to the bending and soldering of the metal structure in the conventional type. Karanxha et al. [35] express the opinion that the buccal inclination of the molars may also be due to the craniofacial rotation of the upper jaw caused by the action of the expander.

The expansion reported by Garib et al. [34] in the area of the first molars is 3.47 mm, which is not significantly less than the 3.64 mm reported by the present authors. This finding indicates that the expansion of the dental arch is within the normal limits.

The expansion of 1.40 mm at the nasal cavity level, facilitated by DDMPTBE, is advantageous in reducing resistance within the nasal airways. Iwasaki et al. [36] also found that Hyrax treatment resulted in a 45.8% reduction in nasal pressure and a 30% reduction in velocity. Hariharan et al. [37] concluded, from a meta-analysis, that RME is an effective therapeutic option for the treatment of OSA in adults and children.

The current study demonstrated that the RME modifies the shape and height of the hard palate, with its height decreasing as a result of expansion. Colino-Gallardo et al. [33] also found that palatal height was reduced by 0.65 ± 0.64 mm, which corresponds to the values of 0.75 ± 0.97 mm proven in the present study. This result leads to the suggestion that the clinically observed reduction in palatal height most likely consists of a true decrease in the palatal height and the widening effect on the palate created by the expanded maxilla. Despite the reduction in the palatal height, this study showed no statistically significant correlation between MxOW and PH. The high rigidity of the appliances, together with the fact that the screw is placed close to the CR, results in a precise force vector leading to almost complete bodily movement with minimal change in the inclination of the molars. This uniform movement (MxOW of 3.64 mm and MxAW of 3.68 mm), which is across the height of the maxillary alveolar ridge, does not indicate any significant deformation in it and thus a change in its height. This may be a probable reason why the increase in MxOW does not contribute to a reduction in PH.

Kavand et al. [38] described no significant difference between tooth- and bone-borne expansion groups, except for the significantly larger increase in buccal inclination of the maxillary right first molar after tooth-borne expansion. Pasqua et al. [18] reported a similar effect in their study and described that the HHE results in more skeletal changes and fewer dental side effects in the studied area of the first premolars. Palatal mini-implants, which are used in HHE, have positive and negative effects. While they improve the skeletal effects achieved by the appliance, they are also associated with negative effects related to their invasive placement and potential loss during the treatment period. They also concluded that the extent of activation affects the degree of change in the nasal section of the skeleton. The present study did not examine the relationship between the number of screw activations and their effect on skeletal changes. The extent of the activations in this case is determined solely by clinical needs.

These negative effects and the absence of a significant expansion difference between the tooth- and bone-borne expanders make the DDMPTBE a viable alternative for RME. DDMPTBE is middle ground between conventional expanders and HHE. Their ability to transfer force directly to the suture and have a screw higher in the palatal vault, similar to HHE, and their ease of manipulation and maintenance, like conventional expanders, combined with a reduced molar inclination, makes DDMPTBE feasible choice for the treatment of MTD. Additionally, their financial cost is less than that of an HHE. However, the greatest advantage of DDMPTBEs is their digital design and planning, which allows the addition of any elements and extensions that are needed, making them perfectly suited and customised for the specific treatment case and goals. The metal printing and laser welding of the appliances ensures their structural accuracy and resistance to corrosion [39].

The action and effectiveness of conventional Hyrax appliances have been extensively studied, even in cases where their design incorporates and relies on temporary molars for anchorage, and the authors found satisfactory skeletal changes [17]. Other authors have also reported the effect of the classic expander on patients with early mixed dentition. They found that the maxillary width and nasal width in the region of the first molars increased by 3.42 ± 0.93 mm (*p* < 0.001) and 2.25 ± 0.77 mm (*p* < 0.001), respectively, after treatment [40]. In the present study, 7 patients had early mixed dentition (Figure 1). The values obtained from the present study are comparable to those found in the aforementioned study, which was conducted on a mixed group of patients with early and late mixed dentition, as well as growing patients with permanent dentition.

The treatment phase with a printed expander leads to normalisation of the molar relationship in the transverse plane. This results in a better growth pattern.

## 5. Limitations

A potential limitation of the study is the relatively small study group of 28 patients.

Thus, the lack of a statistically significant correlation between PH and MxOW might have been influenced by the limited sample size, the age group range and thus be subject to a potential Type II error (false negative). Further research is needed to reach a definitive conclusion on this matter.

The functional changes related to breathing after treatment with a digitally planned expander have not been monitored and are unknown, but the proven skeletal changes in the nasal cavity and maxillary base width suggest the presence of such changes. A study of the functional changes should also include changes in the volume of the upper airways. Measurement of changes in the upper airway volume is the next stage of the team’s research.

The absence of an untreated control group for the purpose of growth comparison is a further limitation of this study. The research team considers that the treatment time with RME is short (around 3 months) and assumes it is unlikely to be significantly affected by active growth, which occurs over a period of 2.5–3 years.

## 6. Conclusions

The use of a DDMPTBE causes skeletal changes, including an increase in the transverse dimension and decrease in the palatal height (−0.75 mm). Although this study did not show a significant correlation between the size of expansion in the MxOW and changes in PH, further research is needed to reach a definitive conclusion on this matter. The DDMPTBE is a feasible treatment option, allowing for precise screw positioning to achieve bodily movement of the teeth and decrease the change in molar inclination.

## Figures and Tables

**Table 1:** Definition of the transverse and vertical variables measured on the coronal slice of the CBCT images.

Variable	Definition
MxOW—Maxillary Occlusal Width	The distance between the central fossa of the upper first molars
MxAW—Maxillary Alveolar Width	The distance between the centres of resistance (CRs) of the upper first molars
MxBW—Maxillary Basal Width	The distance between the intersection of the horizontal line passing through the top of the palate with the maxillary lateral wall
MnAW—Mandibular Alveolar Width	The distance between the centres of resistance (CRs) of the lower first molars
NCW—Nasal Cavity Width	The distance between the widest parts of the lateral nasal walls
HPW—Hard Palatal Width	The distance between the most lateral points of the bony palate
PH—Palatal Height	The perpendicular distance from MxOW to the suture of the hard palate
TDI—Transverse Discrepancy Index	The difference between MxAW1 and MnAW1 TDI < −2.5 mm with no discrepancy when TDI > −2 mm.

**Table 2:** Definition of the first upper molar inclination variables measured on CBCT images.

Variable	Definition
ANG-16—Angulation of tooth 16	The inner angle between the axial tooth plane (defined from F16, the midpoint of the central fossa, to R16, the midpoint between the roots) and the plane that defines the HPW.
ANG-26—Angulation of tooth 26	The inner angle between the axial tooth plane (defined from F26, the midpoint of the central fossa, to R26, the midpoint between the roots) and the plane that defines the HPW.

**Table 3:** Mean changes and standard deviations of the variables before and after treatment.

Variable	*n*	Before Treatment		After Treatment		*p*
		$\bar{X}$	SD	$\bar{X}$	SD	
NCW	28	29.79	2.64	31.19	2.39	<0.001
MxBW	28	60.64	4.09	62.23	3.86	<0.001
MxAW	28	40.73	3.07	44.42	3.19	<0.001
MxOW	28	43.32	3.25	46.95	3.32	<0.001
HPW	28	25.75	3.41	26.94	3.36	<0.001
PH	28	17.48	3.38	16.73	3.27	<0.001

**Table 4:** Changes in the values of the transverse and vertical variables.

Variable	*n*	$\bar{X}$	SD	Min	Max
NCW	28	1.40	0.93	0.12	3.60
MxBW	28	1.59	1.64	−2.42	3.97
MxAW	28	3.68	1.45	−0.37	6.03
MxOW	28	3.64	1.31	0.88	6.43
HPW	28	1.19	1.25	−1.60	4.00
PH	28	−0.75	0.97	−2.76	1.58

**Table 5:** Changes in the inclination of the upper first molars, measured in degrees.

Variable	*n*	$\bar{X}$	SD	Min	Max
ANG-16	28	0.60	6.42	−10.60	18.30
ANG-26	28	2.19	4.51	−6.00	12.60

**Table 6:** Coefficients of the multiple linear regression equation with the dependent variable d_MxOW and independent predictors d_ang16 and d_ang26.

	Unstandardised Coefficients		Standardised Coefficients		
	B	Std. Error	Beta	t	*p*
d_ang16	0.118	0.030	0.576	3.878	0.001
d_ang26	0.138	0.043	0.475	3.198	0.004
Constant	3.262	0.213		15.300	<0.001

## Data Availability

The data that support the findings of this study are available from the corresponding author, G.Y., upon reasonable request.

## Abbreviations

The following abbreviations are used in this manuscript:

MTD	Maxillary transverse deficiency
RME	Rapid maxillary expansion
CAD/CAM	Computer-aided design and computer-aided manufacturing
HHE	Hybrid Hyrax expander
DDMPTBE	Digitally designed metal-printed tooth-borne expanders
CBCT	Cone-beam computed tomography
AI	Artificial intelligence
CR	Centre of resistance
2D	Two-dimensional
TSD	Transverse skeletal discrepancy
CVM	Cervical vertebral maturation
MxOW	Maxillary occlusal width
MxAW	Maxillary alveolar width
MxBW	Maxillary basal width
MnAW	Mandibular alveolar width
NCW	Nasal cavity width
HPW	Hard palatal width
PH	Palatal height
TDI	Transverse discrepancy index
ANG-16	Angulation of tooth 16
ANG-26	Angulation of tooth 26
R^2^	Coefficient of determination
